# Evaluating a Hybrid Circuit Topology for Fault-Ride through in DFIG-Based Wind Turbines

**DOI:** 10.3390/s22239314

**Published:** 2022-11-30

**Authors:** Sarmad Saeed, Rafiq Asghar, Faizan Mehmood, Haider Saleem, Babar Azeem, Zahid Ullah

**Affiliations:** 1 Department of Computer Science, University of Alabama at Birmingham, Birmingham, AL 35294, USA; 2 Department of Electrical Engineering, University of Engineering and Technology, Peshawar 25000, Pakistan; 3 Department of Electrical Engineering, University of Engineering and Technology, Taxila 47050, Pakistan; 4 Department of Electrical and Computer Engineering, University of Alabama at Birmingham, Birmingham, AL 35294, USA; 5 Department of Electrical Energy and Mobility System, Carinthia University of Applied Sciences, 9524 Villach, Austria; 6 Department of Electrical Engineering, UMT Lahore Sialkot Campus, Sialkot 51310, Pakistan

**Keywords:** doubly fed induction generator, fault ride-through, grid faults, renewable energy sources

## Abstract

Large-scale wind power integration has raised concerns about the reliability and stability of power systems. The rotor circuit of a doubly fed induction generator (DFIG) is highly vulnerable to unexpected voltage dips, which can cause considerable electromotive force in the circuit. Consequently, the DFIG must fulfil the fault-ride through (FRT) criteria to ensure the system’s performance and contribute to voltage regulation during severe grid outages. This paper provides a hybrid solution for DFIG wind turbines with FRT capabilities, using both a modified switch-type fault current limiter (MSFTCL) and a direct current (DC) chopper. The proposed system has the merit of keeping the rotor current and the DC-link voltage within the permissible limits, enhancing the FRT capability of generators. Moreover, the boundness of supply voltage into its reference value ensures dynamic stability during symmetric and asymmetric grid failures. Further, electromagnetic torque variations are significantly reduced during fault events. Finally, the performance validation of the proposed scheme is performed in a simulation setup, and the results are compared with the existing sliding mode control (SMC) and proportional-integral (PI) controller-based approaches. The comparison results show that a hybrid strategy with advanced controllers provides superior performance for all critical parameters.

## 1. Introduction

**Figure 1:**
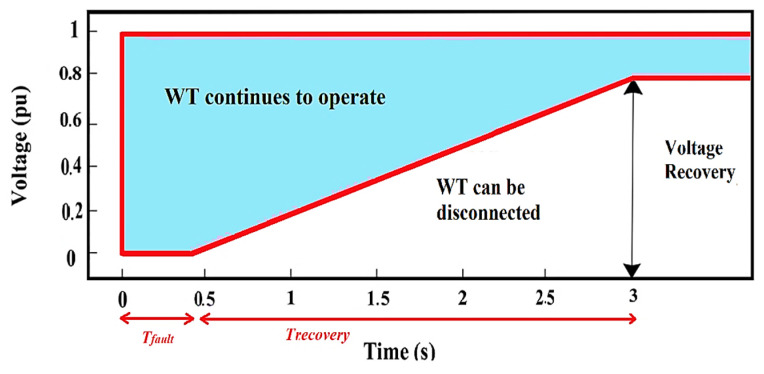
WT system fault ride-through capabilities.

Fault-ride-through (FRT) capability is often considered the most demanding requirement for grid-connected wind turbine (WT) systems. In accordance with these requirements, WTs must continue operating during voltage dips for a defined period and provide reactive power support in both balanced and unbalanced fault conditions [1]. Figure 1 depicts the FRT requirements for the grid-connected WT system [2]. As shown in Figure 1, grid codes require WTs to withstand voltage sags to a specific level, and if the fault lasts for an extended time, WT may be separated from the system to avoid equipment damage. Currently, the doubly fed induction generator (DFIG) is commonly employed to generate power due to its low cost, simplicity of design, and better controllability when used under active and reactive power regulations [3]. Moreover, DFIG allows variable speed operation to capture optimum energy from the wind while minimizing power converter losses. However, since the DFIG stator is directly linked to distribution networks through transformers, any change in grid conditions has a substantial impact on its performance [4]. Particularly, in DFIGs, the FRT problem is exacerbated by the magnetic flux’s inability to keep up with changes in stator voltage and by high slip ratios, which cause considerable voltage and current transients in the rotor circuit [1]. These transients are further exacerbated during unbalanced grid faults due to a negative component in the voltage waveform. In addition, at the start, and after the fault is cleared, the DFIG responses show significant undamped oscillations [5]. Such oscillation may be detrimental to the drivetrain and power electronic converters.

The existing literature has three practical approaches for increasing the FRT capabilities of DFIG-based WTs, including using protective devices, using reactive power injection equipment, and modifying the converter switching frequency [6]. A crowbar circuit has been developed to safeguard the RSC from excessive current during grid disturbances [7]. However, owing to the significant power loss and the conversion of the DFIG to a squirrel induction generator, its application in FRT has been limited [4]. In [8], an energy storage system is proposed to control the DC-link voltage, but it requires RSC to be enough to fulfil the FRT requirements. This problem is alleviated by an improved dynamic voltage restorer (DVR) for FRT enhancement to compensate for voltage fluctuations and disturbances [9]. However, due to the large load and severe harmonic distortion in the inverter’s output, a filter must be added to the DVR output to obtain a pure sine wave.

Similarly, a grid-connected inverter (GCI)-based distributed energy resource is proposed to compensate for voltage fluctuations during grid faults. This control strategy eliminates both active–reactive power oscillations and DC-link voltage oscillations [10,11]. Flexible alternating current transmission system (FACTS) devices are often employed to enhance the FRT capabilities of DFIG-based WTs. For instance, a modified static synchronous compensator (STATCOM) is developed to provide reactive power support and improve power quality in solar and wind-integrated power systems [12]. However, this approach poses the risk of damaging or disconnecting the inverter in the event of a grid fault, and its adoption increases network complexity and costs. In [13], several types of fault current limiters (FCLs) are described for DFIG-based WTs under various configurations but with the drawbacks of non-uniform material heating, inductor saturation, and design complexity. The hybrid techniques for increasing the reliability of the DFIG WT system are given in [14,15]. Specifically, the reliability of the DFIG WT system is improved using dynamic braking resistors (SDBR) and a DC-Chopper [16]. However, the results show that the current and voltage waveforms exhibit significant fluctuations throughout the fault duration. In [17], the FRT capabilities of the WTs system are improved using vector-control techniques that rely on a PI controller. Similarly, DFIG-based WTs may also benefit from using nonlinear controllers based on model parameters and can maintain FRT capability across various operational situations. The nonlinear backstepping control method to increase the DFIG’s FRT capabilities during a network failure is presented in [15]. However, it does not guarantee robustness against uncertainties. The FRT support of DFIG-WTs using the SMC is discussed in [18], which has the merit of accurately managing both the active and the reactive power.

This article presents a novel hybrid approach, combining the modified-switch type fault current limiter (MSFTCL) and direct current (DC) chopper to improve the FRT capability of DFIG wind turbines. Asymmetrical and symmetrical grid faults have been considered for the proposed FRT system. The hybrid system is simpler to implement and can regulate reactive power while maintaining a constant DC-link voltage during fault conditions. Moreover, rotor current oscillations are reduced significantly, protecting the power converters and DC link capacitors during severe grid faults. Additionally, the proposed system reduces stress on the DFIG by maintaining electromagnetic torque magnitude and oscillation within acceptable limits. Both SMC and PI controllers were used for validation purposes, and the results were compared with reference values and without the proposed circuitry. Simulation results indicate that the hybrid strategy provides superior performance for all critical parameters with the advanced controller.

The remainder of our paper is arranged as follows: Section 2 examines wind turbine modelling and control mechanisms for the converters. Section 3 explains the detailed architecture of the proposed system and control schemes. Section 4 presents the simulation findings for SMC and PI controllers employed on the proposed hybrid circuit, while Section 5 presents some concluding remarks.

## 2. Modeling of Wind Energy Conversion System

This section presents detailed modelling of the wind energy conversion system, including the modelling of a doubly fed induction generator, rotor side, and grid side converter.

### 2.1. Wind Energy Conversion System Description

A WECS is an integrated system that combines aerodynamics, automotive, structural, and computational technologies [19]. The WECS system employs a turbine to convert wind kinetic energy into mechanical energy that may be used to generate electricity. The following equations can determine the mechanical power generated by the WTs:

(1) $$\frac{dm}{dt}=\rho A\frac{dx}{dt}$$

(2) $$\frac{dK\cdot E}{dt}=\frac{1}{2}\rho A\;V^{3}$$

(3) $$P_{m}=\frac{1}{2}\rho AV^{3}Cp\left(\lambda ,\delta \right)$$

where A, ρ, and δ are the swept area, air density, and pitch angle of WTs, respectively. The essential design parameters include the tip speed λ and power coefficient *C_p_* for total power extraction of WTs [20]. The *λ* can be described as the turbine’s tangent speed ratio to the actual wind speed, and Cp is the ratio of the actual power generated to the maximum wind power accessible at the blades. More detailed insight into the modeling of WECS can be found in [21].

(4) $$\lambda =\frac{\omega R}{V}$$

(5) $$C_{p}\left(\lambda ,\delta \right)=B_{1}\left(\frac{B_{2}}{\lambda_{j}}-B_{3}\delta -B_{4}\right)e^{-\frac{B_{5}}{\lambda_{j}}}+B_{6}\lambda$$

where $B_{1}=571.6\times 10^{-3},\;B_{2}=116,\;B_{3}=0.4,\;B_{4}=5,\;B_{5}=21,\;B_{6}=6.8\times 10^{-3}$ and $\frac{1}{\lambda_{j}}=\frac{1}{\lambda +0.08\delta }-\frac{0.035}{\delta^{3}+1}$. The estimated power efficiency of WTs, represented by Betz’s or Lanchester’s limit, is 0.593 [21]. Therefore, it is important to calibrate the speed at the turbine’s shaft to achieve the maximum tip speed and power coefficient to maximize active power generation. To capture the maximum wind energy, larger turbine blades are often used to increase the air’s density and covered area. It is also important to note that air density is influenced by temperature, height, and humidity. Such factor variations may cause the density to vary by as much as 10% in certain situations. Further, since the output power is proportional to the speed cube, even modest changes in wind velocity may result in a considerable shift in total wattage.

### 2.2. Modelling of Doubly Fed Induction Generator

#### 2.2.1. Modelling of DFIG under Balanced Conditions

DFIG’s control is more sophisticated because it can operate in sub-synchronous and super-synchronous regions compared to other induction generators. Since the rotor winding rotates in a periodic pattern, that can change the stator angle, causing a variation of inductance matrix values [22]. Consequently, the current and voltage will have time-dependent values, making it difficult to solve these equations. Hence, the dqo transformation is applied to obtain the static inductance matrices. Now, assuming $T_{dqo}\left(\beta \right)$ is a rotating matrix that transforms abc signals into dqo frames, the voltage equations for the DFIG in the dqo frames are as follows:

(6) $$T_{dqo}\left(\beta \right)=\sqrt{\frac{3\;}{2}}\left[\begin{array}{ccc} \;cos\beta & \;cos\left(\beta -\frac{2\pi }{3}\right) & cos\left(\beta +\frac{2\pi }{3}\right)\\ -sin\beta & -sin(\beta -\frac{2\pi }{3}) & sin(\beta +\frac{2\pi }{3})\;\\ \;\sqrt{\frac{1}{2}} & \;\sqrt{\frac{1}{2}} & \;\sqrt{\frac{1}{2}} \end{array}\right]$$

A generalized transformation can be performed using the following equation.

(7) $${\vec{f}}_{dqo}=T_{dqo}\left(\beta \right){\vec{f}}_{abc}$$

where ${\vec{f}}_{abc}=\left[\begin{array}{c} fa\\ fb\\ fc \end{array}\right]$ and ${\vec{f}}_{dqo}=\left[\begin{array}{c} f_{d}\\ f_{q}\\ f_{0} \end{array}\right]$. In the case of abc signal-balanced quantities, it becomes

(8) $$f_{0}=\sqrt{3}/2\left(f_{a}+f_{b}+f_{c}\right)=0$$

The DFIG’s voltage equations in the dqo frames are as follows:

(9) $${\vec{v}}_{dqs}=R_{s}{\vec{i}}_{dqs}+T_{dqo}\left(\beta \right)\frac{d}{dt}\left(T_{dqo}{\left(\beta \right)}^{-1}{\vec{\varphi }}_{dqs}\right)$$

(10) $${\vec{v}}_{dqr}=R_{s}{\vec{i}}_{dqr}+T_{dqo}\left(\beta \right)\frac{d}{dt}\left(T_{dqo}{\left(\beta \right)}^{-1}{\vec{\varphi }}_{dqr}\right)$$

(11) $${\vec{\varphi }}_{dqs}=T_{dqo}\left(\beta \right)L_{s}T_{dqo}{\left(\beta \right)}^{-1}{\vec{i}}_{dqs}+T_{dqo}\left(\beta \right)L_{sr}\left(T_{dqo}{\left(\beta \right)}^{-1}\right){\vec{i}}_{dqs}$$

(12) $${\vec{\varphi }}_{dqr}=T_{dqo}\left(\beta \right)L_{r}T_{dqo}{\left(\beta \right)}^{-1}{\vec{i}}_{dqr}+T_{dqo}\left(\beta \right)L_{sr}{}^{T}\left(T_{dqo}{\left(\beta \right)}^{-1}\right){\vec{i}}_{dqr}$$

The voltage and flux equations can be simplified by [22,23], which gives

(13) $$v_{ds}=R_{s}i_{ds}+\frac{d\varphi_{ds}}{dt}-\omega \varphi_{qs}$$

(14) $$v_{qs}=R_{s}i_{qs}+\frac{d\varphi_{qs}}{dt}+\omega \varphi_{ds}$$

(15) $$v_{dr}=R_{r}i_{dr}+\frac{d\varphi_{dr}}{dt}-(\omega -\omega_{r})\varphi_{qr}$$

(16) $$v_{qr}=R_{r}i_{qr}+\frac{d\varphi_{qr}}{dt}+(\omega -\omega_{r})\varphi_{dr}$$

The impedance matrices without a zero component can be represented as follows [22]:

(17) $$\varphi_{ds}=(L_{\sigma s}+\frac{3}{2}L_{M})i_{ds}+\frac{3}{2}L_{M}i_{dr}$$

(18) $$\varphi_{qs}=(L_{\sigma s}+\frac{3}{2}L_{M})i_{qs}+\frac{3}{2}L_{M}i_{qr}$$

(19) $$\varphi_{dr}=(L_{\sigma s}+\frac{3}{2}L_{M})i_{dr}+\frac{3}{2}L_{M}i_{ds}$$

(20) $$\varphi_{qr}=(L_{\sigma s}+\frac{3}{2}L_{M})i_{qr}+\frac{3}{2}L_{M}i_{qs}$$

The transformation removes the time dependence for impedance and reduces the model’s complications. Since the flux linkages per second are required to solve the electrical equations, their dependency on the synchronous speed must also be considered [22]. Conversely, the electrical equations are calculated using the flux linkage per second Ψ=φω as follows [21]:

(21) $$\Psi_{ds}=-X_{s}\;i_{ds}+X_{M}\;i_{dr}$$

(22) $$\Psi_{qs}=-X_{s}\;i_{qs}+X_{M}\;i_{qr}$$

(23) $$\Psi_{dr}=-X_{r}\;i_{dr}+X_{M}\;i_{ds}$$

(24) $$\Psi_{qr}=-X_{r}\;i_{qr}+X_{M}\;i_{qs}$$

We calculated the reactance $\left(X_{s}\;,X_{r},X_{M}\right)$ as follows:

(25) $$X_{s}=\omega \left(L_{\sigma s}+\frac{3}{2}L_{M}\right)$$

(26) $$X_{r}=\omega \left(L_{\sigma r}+\frac{3}{2}L_{M}\right)$$

(27) $$X_{M}=\frac{3}{2}\omega L_{M}$$

Similarly, the stator and rotor’s active and reactive power consumption can be determined as follows [24]:

(28) $$P_{s}=\frac{3}{2}\left(v_{ds}i_{ds}+v_{qs}i_{qs}\right)$$

(29) $$P_{r}=\frac{3}{2}\left(v_{dr}i_{dr}+v_{qr}i_{qr}\right)$$

(30) $$Q_{s}=\frac{3}{2}\left(v_{qs}i_{ds}-v_{ds}i_{qs}\right)$$

(31) $$Q_{r}=\frac{3}{2}\left(v_{qr}i_{dr}-v_{dr}i_{qr}\right)$$

Considering that Tm=Te, the electromagnetic torque Te and the input torque Tm combine to form a homogeneous steady-state system.

(32) $$T_{e}=\frac{3}{2}pL_{M}\left(i_{qs}i_{dr}-i_{ds}i_{qr}\right)$$

where p denotes the poles pair.

#### 2.2.2. Behavior of the DFIG under Unbalanced Conditions

Normally, the stator flux has a constant magnitude$\;\varphi_{s}$ and rotates at synchronous speed ω, whereas the back emf is relatively small. However, the situation changes adequately during voltage dips due to a change in stator flux magnitude, which directly influences the rotor emf. This causes significant transients in both the voltage and current waveforms [1]. Additionally, the DFIG outputs exhibit considerable undamped oscillations at the start and after the fault is cleared, which may be detrimental to the drivetrain and power electronic converters [5]. We considered the synchronous reference frame while estimating the impact of unbalanced grid conditions. The following equations can be used to compute the stator and rotor voltages and fluxes in the d-q reference [25]:

(33) $$v_{sdq}=R_{s}i_{sdq}+j\omega \Psi_{sdq}+\frac{1}{\omega_{a}}\frac{d\Psi_{sdq}}{dt}\;$$

(34) $$v_{rdq}=R_{r}i_{rdq}+j\omega_{r}\Psi_{rdq}+\frac{1}{\omega_{a}}\frac{d\Psi_{rdq}}{dt}\;$$

(35) $$\Psi_{sdq}=L_{s}i_{sdq}+L_{M}i_{rdq}\;$$

(36) $$\Psi_{rdq}=L_{r}i_{rdq}+L_{M}i_{sdq}\;$$

The rotor voltage is calculated using the rotor current and flux from Equations (34)–(36).

(37) $$v_{rdq}=R_{t}i_{rdq}+j\omega_{r}L_{t}i_{rdq}+\frac{L_{t}}{\omega_{a}}\frac{di_{rdq}}{dt}+E_{b}$$

$R_{t}$, $L_{t}$ are defined as the transient resistance and inductance, respectively, as follows:

(38) $$R_{t}=(R_{r}L_{s}{}^{2}+R_{s}L_{M}{}^{2})/L_{s}{}^{2}$$

(39) $$L_{t}=((L_{s}L_{r}-L_{M}{}^{2})/L_{s}$$

In Equation (37), $E_{b}$ represents the back emf, which significantly affects both the rotor current and the Dc-link voltage. The magnitude of Eb is determined by the stator flux and voltage and may be computed as follows:

(40) $$E_{b}=\frac{L_{M}}{L_{s}}\left(v_{rdq}-j\omega_{r}\Psi_{sdq}-\frac{R_{s}}{L_{s}}\Psi_{sdq}\right)$$

When a grid fault occurs, the stator voltage rapidly decreases in magnitude, but stator flux does not follow the voltage change [26]. Consequently, the rotor circuit experiences an extra instantaneous flux to maintain stator flux continuity. Hence, the stator flux during a voltage dip can be calculated as follows:

(41) $$\Psi_{sdq}\left(t\right)=\Psi_{s}{}^{+}+\Psi_{s}{}^{-}e^{-j2\omega_{b}t}+(\Psi_{in}{}^{+}-\Psi_{s}{}^{+}-\Psi_{s}{}^{-})e^{-\sigma t}e^{-{j\omega }_{b}t}$$

The symbols +, −, and in represent the positive sequence, negative sequence, and initial value, respectively. The first and second parts correspond to the steady-state positive and negative sequence fluxes, respectively, while the third describes the natural flux. Each of the three fluxes affects the magnitude of the induced back-emf in the rotor circuit. The rotor back-emf caused by these fluxes may be expressed as

(42) $$E_{b}=j\frac{L_{M}}{L_{s}}\left(s\Psi_{s}{}^{+}-\left(2-s\right)\Psi_{s}{}^{-}e^{-j2\omega_{b}t}-\omega_{r}(\Psi_{in}{}^{+}-\Psi_{s}{}^{+}-\Psi_{s}{}^{-}\right)e^{-\sigma t}e^{-{j\omega }_{b}t}$$

According to the above equation, the negative sequence component oscillates at double the grid’s frequency. Therefore, catastrophic unbalanced voltage sags with large percentages of negative sequence voltage may result in large back-emfs.

### 2.3. Rotor Side Converter Control

The RSC must simultaneously regulate the generator in sub-synchronous and super-synchronous states, requiring a more advanced regulating system than a conventional grid-side converter (GSC) control system. Further, such converters must be able to respond to the output power patterns of WTs, thus managing the system’s power factor. Aside from these considerations, the primary objective of RSC is to maintain a constant rotor speed regardless of variations in wind speed. It is possible to control the power flows in the DFIG with the rotor current content [22]. The voltage equations$\;v_{ar}$, $v_{br}$, and $v_{cr}$ are described as follows:

(43) $$v_{ar}=v_{r}sin(\omega t+\phi_{gr})$$

(44) $$v_{br}=v_{r}sin(\omega t+\frac{2\pi }{3}\phi_{gr})\;$$

(45) $$v_{cr}=v_{r}sin(\omega t-\frac{2\pi }{3}\phi_{gr})$$

This study employs voltage-oriented control (VOC) for controlling the RSC. The rotor current’s dr and dq components control the amount of active and reactive power, which can be expressed as $P_{s}\propto i_{dr}$ and $Q_{s}\propto i_{qr}$. With VOC, the d-axis of the synchronous reference frame (SRF) aligns with the stator-voltage vector. Therefore, $v_{qs}=0$, $v_{ds}=v_{s}.$ Equation (21) can be simplified as follows [21]:

(46) $$0=-X_{s}\;i_{ds}+X_{M}\;i_{dr}$$

(47) $$\;i_{ds}=\frac{X_{M}}{X_{s}}\;i_{dr}$$

(48) $$\Psi_{qs}=\frac{v_{s}}{\omega_{s}}=\left|\Psi_{s}\right|\;$$

(49) $$\left|\Psi_{s}\right|=X_{s}\;i_{qs}+X_{M}\;i_{qr}$$

(50) $$i_{qs}=\frac{\left|\Psi_{s}\right|}{X_{s}}-\frac{X_{M}}{X_{s}}\;i_{qr}$$

Using the values of $i_{q}$ and $i_{d}$ as inputs, the stator powers can be estimated as follows:

(51) $$P_{\;s}=-\frac{3}{2}\;\frac{X_{M}}{X_{s}}v_{s}\;i_{dr}$$

(52) $$\;Q_{\;s}=-\frac{3}{2}\;(\frac{\left|\psi_{s}\right|}{X_{s}}-\frac{X_{M}}{X_{s}}\;i_{qr})v_{s}$$

According to the above equations, both powers are handled by varying the rotor d or q currents.

### 2.4. Grid-Side Converter Control

The DC-link voltage oscillates due to active power fluctuations, forcing capacitors to charge and discharge frequently. These voltage oscillations shorten the life of the capacitor and cause power losses. Furthermore, such oscillations generate harmonic components on the grid, which may cause excessive current strains, power quality issues, and significant power losses [10]. Hence, the primary function of the GSC is to maintain a constant DC link voltage regardless of variations in wind speed or grid disturbance. In addition, it also supports the grid voltage with reactive power. Transient currents may occur due to the phase difference between the GSC and the grid voltage. Such transients are prevented by synchronizing GSCs with the grid. Hence, it will provide a steady-state alternative current to the rotor circuit to ensure flux stability. The study employed VOC to control GSCs and regulate the active/reactive power of GSCs and grids. The voltage equations can be derived as [21],

(53) $$v_{gd}=Ri_{gd}+L\frac{di_{gd}}{dt}+\omega Li_{gq}-U_{cd}$$

(54) $$v_{gq}=Ri_{gq}+L\frac{di_{gq}}{dt}+\omega Li_{gd}+v_{cq}$$

The subscripts g and c represent the voltage and current of the grid/converter, respectively. The power equations of the GSC are determined as follows:

(55) $$P_{g}=\frac{3}{2}\left(v_{gd}i_{gd}+v_{gq}i_{gq}\right)$$

(56) $$Q_{g}=-\frac{3}{2}(v_{gd}i_{gq}+v_{gq}i_{gd})\;$$

As $v_{gd}=\left|{\vec{v}}_{s}\right|\;\mathrm{and}\;v_{gq}=0$, Equation (45) becomes,

(57) $$P_{g}=\frac{3}{2}\left(v_{gd}i_{gd}\right)$$

(58) $$Q_{g}=-\frac{3}{2}\left(v_{gd}i_{gq}\right)$$

Conversely, the DC-Link energy is denoted by the following expression:

(59) $$W_{dc}=\int Pdt=\frac{1}{2}C_{dc}U_{dc}{}^{2}=P_{g}-P_{r}$$

**Figure 2:**
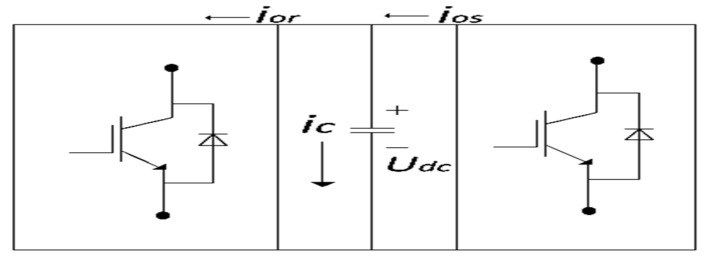
DC-Link model.

Figure 2 demonstrates an appropriate design for a DC-Link, which exhibits the multiple variables of the system. Current flowing through converters can be calculated as follows:

(60) $$\;U_{dc}i_{os}=v_{gd}i_{gd}$$

(61) $$v_{gd}=\frac{1}{2\sqrt{2}}m_{1}U_{dc}$$

(62) $$i_{os}=\frac{1}{2\sqrt{2}}m_{1}i_{gd}$$

(63) $$C\frac{dU_{dc}}{dt}=\;i_{os}-i_{or}$$

In this equation,$\;m_{1},$ $\;i_{or},\;\;i_{os}$ represent the modulation index, rotor-dc link current, and grid-dc link current, respectively.

(64) $$\;i_{gd}=\frac{2\sqrt{2}}{3m_{1}}C\frac{dU_{dc}}{dt}$$

This leads to the conclusion that the voltage control is susceptible to the converter’s current $\;i_{gd}$.

## 3. Fault Ride through Control Circuit Design

### 3.1. Proposed Circuit Topology

**Figure 3:**
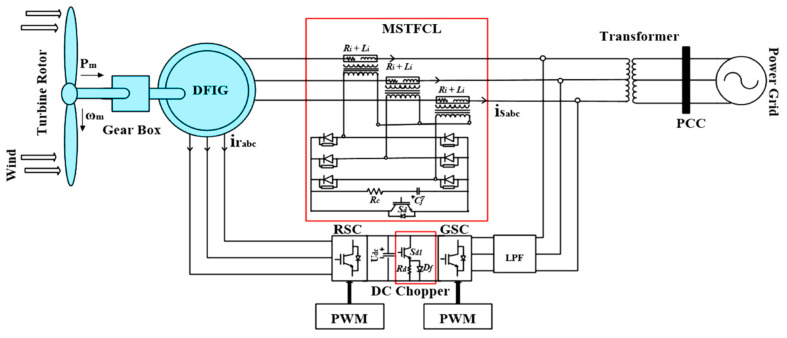
Schematic of proposed system.

The single-line diagram used in this study is shown in Figure 3. An MSTFCL is coupled with the DFIG to prevent excessive currents and back-EMFs during symmetrical and asymmetrical fault conditions. The system also includes a DC-chopper, which protects the DC-Link against voltage surges during grid disturbances. In this configuration, integrated 9 MW DFIG-based WTs are connected to an infinite bus with a rated voltage of 575 V. The grid also contains a 150 MVA synchronous generator with an output voltage of 13.8 kV and a source frequency of 60 Hz. The DFIG output voltage is increased from 575 V to 25 kV using step-up transformers, and the point of common coupling (PCC) voltage is adjusted using another step-up transformer. The detailed specifications of the suggested scheme are shown in Table 1.

#### 3.1.1. Modified Switch Type Fault Current Limiter Design

The components of the MSTFCL are depicted in Figure 3: A snubber circuit ($C_{f},\;R_{c}$), a limiting inductor ($L_{i}$), a limiting resistor ($R_{i}$), a bridge circuit ($D_{1}$*–*$D_{6}$), and a semiconductor switch ($S_{d}$). The semiconductor switch ($S_{d}$) is normally open, bypassing the limiting inductor and resistor. When a grid fault is detected, the$\;S_{d}$ is turned off, and the snubber capacitor initially performs two functions: First, it stores excess energy, and second, it protects the $S_{d}$ against voltage spikes. Using Kirchhoff’s voltage law (KVL), the stator voltage $v_{s}=v_{m}sin(\omega t+\vartheta )$ can be represented as follows:

(65) $$v_{m}sin(\omega t+\vartheta )=R_{T}I_{c}\left(t\right)+L_{T}\frac{dI_{c}\left(t\right)}{dt}+\frac{1}{C_{f}}\;\int I_{c}\left(t\right)dt$$

In Equation (55), $R_{T}$ represents the combined stator resistance ($R_{s}$) and snubber circuit resistance ($R_{c}$) and $L_{T}$ represents the combined stator ($L_{s}$*)* and parasitic inductance ($L_{p}$). $I_{c}\left(t\right)$ denotes the capacitor current. When the initial condition is known, the voltage of the snubber capacitor $V_{c}\left(t\right)$ can be determined as

(66) $$v_{c}\left(t\right)=\frac{1}{C_{f}}\;\int I_{c}\left(t\right)dt+v_{i}$$

Similarly, the current through the capacitor can be determined as

(67) $$I_{c}\left(t\right)=\frac{v_{m}}{Z_{T}}sin(\omega t+\vartheta -\emptyset )+I_{n\_r}\left(t\right)$$

where $I_{n\_r}\left(t\right)$ and $Z_{T}$ denote the system’s natural response and total impedance, respectively. Depending on the system’s resistance, capacitance, and inductance values, the above-indicated system may have underdamped, overdamped, or critically damped natural responses. In this situation, the snubber resistor is eleven times larger than the rotor resistor, resulting in an overdamped system response; hence, the system’s natural response can be expressed as

(68) $$I_{n\_r}\left(t\right)=A_{1}e^{s1\left(t-t_{i}\right)}+A_{2}e^{s2\left(t-t_{i}\right)}$$

(69) $$Z_{T}={\left(R_{T}{}^{2}+(\omega L_{T}-\frac{1}{\omega C_{f}})\right)}^{1/2}$$

(70) $$\emptyset =\tan(\omega L_{T}-\frac{1}{\omega C_{f}})/R_{T}$$

where $s_{1},s_{2}$ denote natural frequencies, and after solving Equation (68), the natural frequencies can be determined as follows:

(71) $$s_{1,2}=\frac{-R_{T}\pm \sqrt{R_{T}{}^{2}-4L_{T}/C_{f}}}{2L_{T}}$$

When Equation (71) is compared to the generalized equation, the damping coefficient (β) and natural frequency ($\omega_{o}$) may be represented as [27]

(72) s1,2=−β±β2−ωo2, β=RT2LT, ωo=1LTCf

Given the initial conditions, we can calculate $A_{1}$ and $A_{2}$ as follows [27]:

(73) $$A_{1}=-\frac{v_{m}}{2Z_{T}\sqrt{\beta^{2}-\omega_{o}{}^{2}}}\left[s_{2}sin(\omega t+\vartheta -\emptyset \right)+\omega cos(\omega t+\vartheta -\emptyset )]-\frac{v_{m}sin(\omega t+\vartheta )-v_{i}}{2L_{T}\sqrt{\beta^{2}-\omega_{o}{}^{2}}}$$

(74) $$A_{2}=-\frac{v_{m}}{2Z_{T}\sqrt{\beta^{2}-\omega_{o}{}^{2}}}\left[s_{2}sin(\omega t+\vartheta -\emptyset \right)+\omega cos(\omega t+\vartheta -\emptyset )]-\left(\frac{v_{m}sin(\omega t+\vartheta )-v_{i}}{2L_{T}\sqrt{\beta^{2}-\omega_{o}{}^{2}}}\right)$$

When the $C_{f}$ voltage reaches its limit, the limiting inductor and resistor are entirely inserted into the circuit to cut off the current through the diode bridge. The limiting inductor and the resistor, which relieved the strain on the DFIG WTs, limited the current fault level. During both symmetrical and asymmetrical faults, the rotor voltage reaches its maximum amplitude as follows:

(75) $$v_{r\_sym\_max}=L_{m}/L_{T1}\times v_{s}\left(s\left(1-d\right)+\left(1-s\right)d\right)$$

(76) $$v_{r\_asy\_max}=L_{m}/L_{T1}\times v_{s}s\left(0.5-\left(1.5-2/s\right)\right)$$

where s*,* d, and$\;L_{m}$ represent the generator slip, depth of voltage dip, and mutual inductance, respectively. $L_{T1}$ represents the combined stator ($L_{s}$) and limiting inductance ($L_{i}$). $L_{T1}$ exceeds $L_{m},\;$so the maximum voltage can be maintained within acceptable limits.

#### 3.1.2. DC Chopper Design

The DC-Chopper is a protective device that uses a resistor to short-circuit the DC link if the grid voltage drops. Figure 2 depicts the parallel connection of the DC copper at the DC link, which comprises a chopper resistor ($R_{d})$, a freewheeling diode $\left(D_{f}\right)$, and a semiconductor switch $(S_{d1}$). When the switch $S_{d1}$ is closed, the $D_{f}$ protects the $S_{d1}$ from high-voltage surges. During regular operation, the switch is open to bypass the resistor; however, when an excessive voltage is detected, the switch closes, enabling the additional energy to be dissipated across the resistor and thereby limiting the voltage at DC-Link. The DC chopper regulates the fault current through the chopper resistor by controlling the duty cycle D of the semiconductor switch. When the voltage across the DC link exceeds the threshold voltage ($U_{DC-T}$) during a fault condition, the equation for D can be expressed as [28].

(77) D=(kpce+kices ) (VDC−I−UDC−T), 0 ≤ D ≤ 1

where $k_{pce}$ and $k_{ice}$ are the PI controller coefficients.$\;V_{DC-I}$ is the instantaneous DC link voltage across the capacitor.

### 3.2. Control Scheme Design

#### 3.2.1. SMC Controller

SMC is a more advanced control technology that provides a robust method for obtaining the desired performance even when the system is subject to disturbances and uncertainty [29]. The high-gain feedback of SMC reduces the complexity of the system when compared to nonlinear techniques. Reduced state space designs may provide resilience, ease of installation, and efficient elimination of perturbations, all desirable characteristics. Two stages are involved in developing SMC, such as (a) designing the sliding surface and formulating it and (b) refining the control law. As part of the design process, a hypersurface is created first to enumerate requirements and then to drive the system state trajectory. Following this, a control law is developed to optimize the system’s trajectory and maintain its position on the hypersurface. The state space equations can be expressed in the following way [30]

(78) $$\dot{x}=A\left(x,t\right)+B\left(x,t\right)\cdot u\left(x,t\right)$$

where x, A(x,t)B(x,t) and u denote the state vector, two nonlinear random functions, and the control vector, respectively. Equation (68) can be used to define the sliding surface or hypersurface

(79) $$SS\;\left(x,t\right)={\left(\frac{d}{dt}+\tau \right)}^{n-1}\cdot e$$

where $e=x_{a}-x$*,* $x_{a}=\left[x_{a},{\dot{x}}_{a},\ldots x_{a}{}^{n-1}\right]{\;}^{T}$ are anticipated vectors, $x={\left[x,x,\ldots x^{n-1}\right]}^{T}$ is the state vector, and $e={\left[e,\dot{e},\ldots e^{n-1}\right]}^{T}$ is the error vector. Lyapunov’s solution satisfies the convergence criterion by maintaining a stable and attractive surface.

(80) $$V=\frac{1}{2}S{\left(x\right)}^{2}$$

The Lyapunov principle predicts that if V is less than one, the system’s path will be attracted to the hypersurface, remaining until it asymptotically returns to its initial position.

(81) $$S\left(x\right)\dot{S}\left(x\right)\leq 0$$

Accordingly, the control law that meets the antecedent requirements is:

(82) $$\{\begin{array}{c} u=u^{a}+u^{c}\\ u^{c}=-k_{p}\cdot sgn\left(\sigma \left(x,t\right)\right) \end{array}$$

where $u,\;u^{a},u^{c},\;\mathrm{and}\;k_{p}$ denote the control vector, appropriate control vector, correction factor, and controller gain. When the system parameters have reached the hypersurface, the corresponding control maintains its position.

(83) $$sgn\left(\emptyset \right)=\{\begin{array}{c} 1\;\emptyset >0\\ 0\;\emptyset =0\\ -1\;\emptyset <0 \end{array}$$

The controller represented by Equation (72) is robust and waterproof to parameter changes and perturbations, but the sgn function, which is used, causes chattering along the hypersurface. Such significant input variations may be avoided by setting up a border condition with the width of *ϵ*. Hence, Equation (72) may be rewritten by substituting sat(σ(t)/ϵ for sgn(σ(t)/ϵ.

(84) $$u^{c}=-k_{p}\cdot sat\left(\sigma \left(x,t\right)\right)$$

where ϵ>0,

(85) $$sat\left(\emptyset \right)=\{\begin{array}{c} sgn\left(\emptyset \right)\;\emptyset \geq 0\\ \;\emptyset \;\emptyset <0 \end{array}$$

DFIG is regulated by surfaces that measure the difference between the intended and actual signal transmitted.

#### 3.2.2. PI Controller

Even though novel control approaches have been developed from a conventional PI controller, it continues to have a wide variety of applications in power systems, particularly WTs, provided the PI-controlled variables are appropriately tuned [31]. Hence, it is a popular choice for industrial and computational modeling because of its simplicity and long life expectancy [32]. Model estimations, pattern designing, and parameter modification are necessary for a PI controller to analyze the output signals accurately. A model estimate involves altering a system’s output values at a specified sample frequency and then utilizing the results to produce an accurate prototype. Following model identification, the controller design should be optimized to reduce chattering issues while demonstrating resilience to load disturbances and parameter changes.

(86) $$v\left(t\right)=v_{p}\left(t\right)+v_{i}\left(t\right)$$

(87) $$v\left(t\right)=k_{p}\;e\left(t\right)+\frac{k_{i}}{T_{i}}\;\int e\left(t\right)dt$$

(88) e(t)=SP−PV

where e(t), SP, and PV are the error signal, setpoint, and process variable, respectively. The gain of a PI controller can be determined by applying a Laplace transform on Equation (77).

(89) $$V\left(s\right)=k_{p}\;E\left(s\right)+k_{i}\frac{E\left(s\right)}{s}$$

(90) $$V\left(s\right)=E\left(s\right)\;\left(\;k_{p}+\frac{k_{i}}{s}\;\right)$$

Notably, the error signal will act as an input, causing the controller’s output to fluctuate. By simplifying Equation (80), we obtain

(91) $$\frac{V\left(s\right)}{E\left(s\right)}=k_{p}\left(\;1+\frac{k_{i}}{k_{p}s}\;\right)$$

(92) $$\frac{V\left(s\right)}{E\left(s\right)}=k_{p}\left(\;1+\frac{1}{\tau_{g}s}\;\right)$$

where $\tau_{g}=\frac{k_{p}}{k_{i}}$ is the controller’s gain.

## 4. Results and Discussion

This part discusses the interaction between DFIG-based WTs and the power system in case of a grid fault. Two scenarios are evaluated to demonstrate the effectiveness of the proposed approach. Based on scenario one, the system is subjected to a balanced three-phase symmetric fault. The outcomes are compared for the reference value without a control scheme, the SMC, and PI controllers. The suggested system is assessed for the line-line-to-ground (LL-G) asymmetrical fault type in scenario two. In both scenarios, grid disturbances are expected to occur on the 30-km transmission line that connects the step-up transformers to the distribution system. The faults had a length of 150 ms, and we assumed that the wind speed remained constant during the entire simulation period. In both scenarios, five essential factors are investigated for symmetrical and asymmetrical faults, and the findings have been compared for the SMC and PI controllers.

### 4.1. Symmetrical Grid Fault

**Figure 4:**
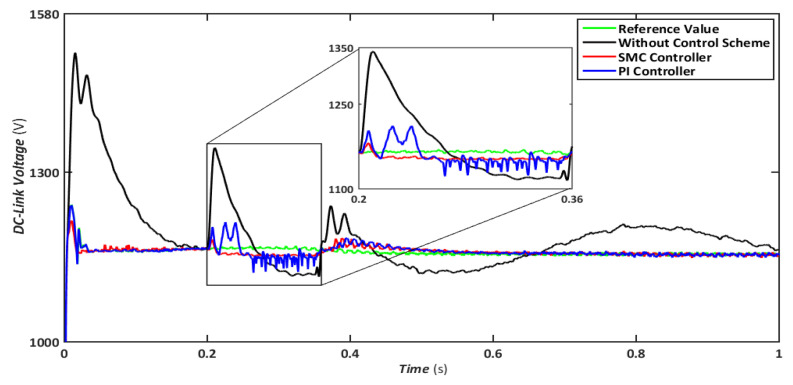
DC link voltage during symmetrical fault.

**Figure 5:**
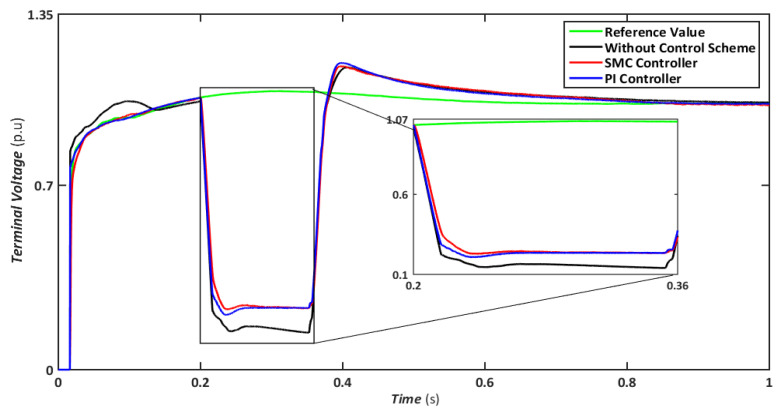
Terminal voltage during symmetrical fault.

**Figure 6:**
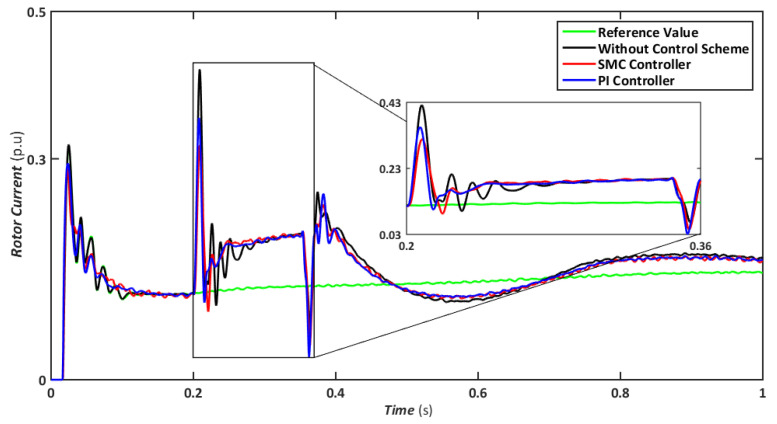
Rotor current during symmetrical fault.

**Figure 7:**
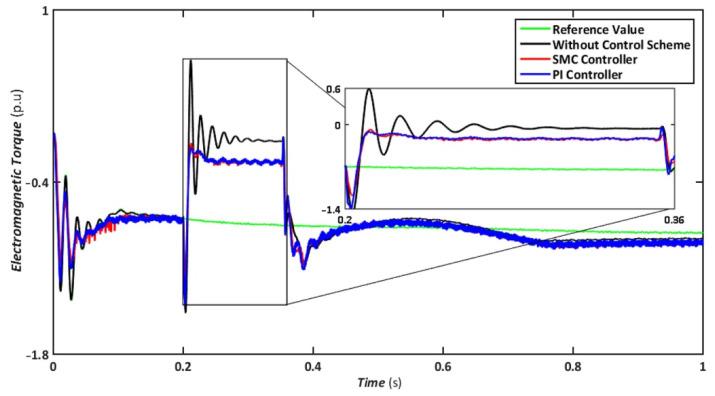
Electromagnetic torque during symmetrical fault.

**Figure 8:**
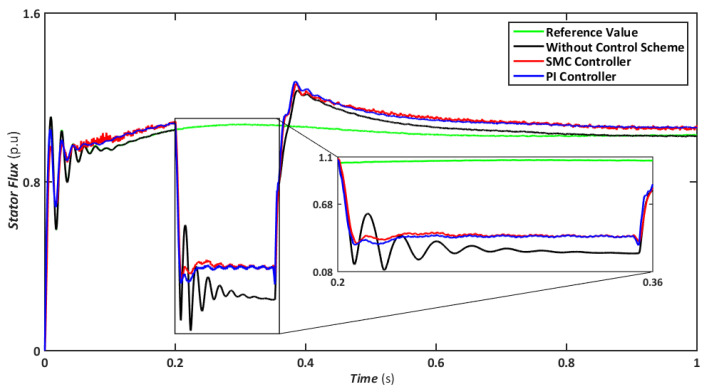
Stator flux during symmetrical fault.

The symmetrical fault is the most catastrophic failure in a power system, in which all phases are shorted to one another or the ground. Even though these disturbances can carry a significant amount of current, they are relatively rare. In this scenario, a three-line-to-ground (LLLG) fault is introduced on the transmission line that runs between the step-up transformers, and the findings are simulated. Figure 4, Figure 5, Figure 6, Figure 7 and Figure 8 compare the simulation results for the reference value, SMC controller, PI controller, and without control schemes, represented by green, red, blue, and black lines, respectively. According to Figure 4, a grid failure caused the DC-Link voltage to rise to 1.8 kV when the SMC controller was applied. However, this voltage stayed unchanged at 1.14 kV throughout the fault and restoration periods. When using the PI controller, the voltage rises to 1.19 kV, and oscillations can be detected up and down in the frequency spectrum. Similarly, both SMC and PI controllers can maintain voltage drops of 78 percent, as shown in Figure 5, demonstrating the effectiveness of DFIG’s reactive power compensation. Even though the rotor current remains equal throughout the restoration time, the proposed SMC-based system exhibits superior control over the PI controller during the fault phase, as seen in Figure 6. Compared to the PI controller, the SMC controller controlled the rotor current to 0.3 pu rather than 0.35 pu. Since the electromagnetic torque was negative in Figure 7, this demonstrates that the DFIG reduces transmission system stresses and improves grid power stability. The findings reveal that the maximum value for SMC was −1.18 pu, whereas the comparable value for the PI controller was −1.4 pu. As can be seen in Figure 8, the SMC controller dominates performance and keeps the stator flux amplitude at approximately 0.32 pu, decreasing the back-EMF influence.

### 4.2. Asymmetrical Grid Fault

**Figure 9:**
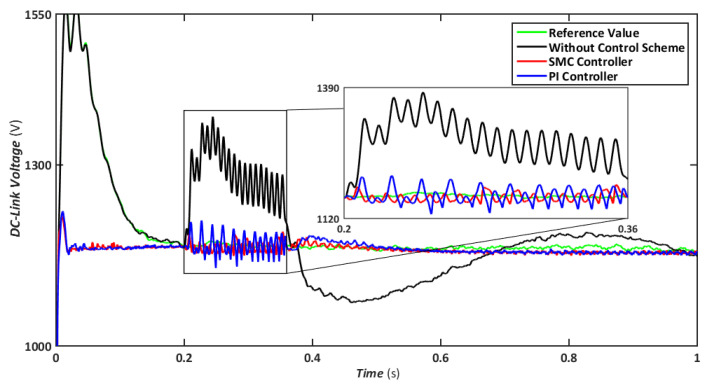
DC link voltage during asymmetrical fault.

**Figure 10:**
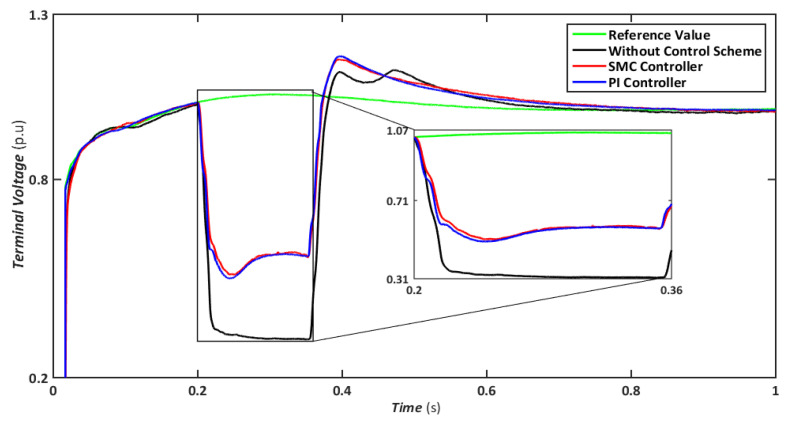
Terminal voltage during asymmetrical fault.

**Figure 11:**
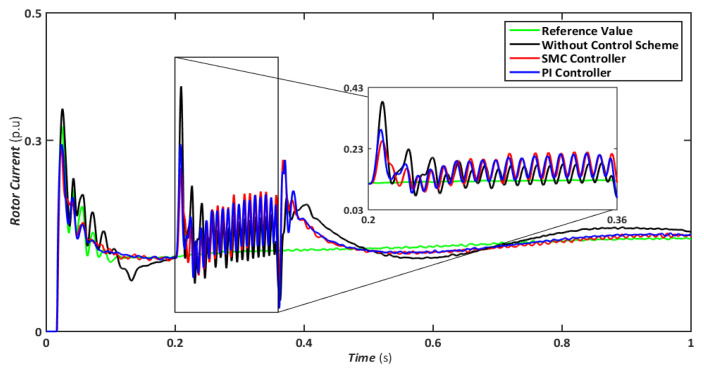
Rotor current during asymmetrical fault.

**Figure 12:**
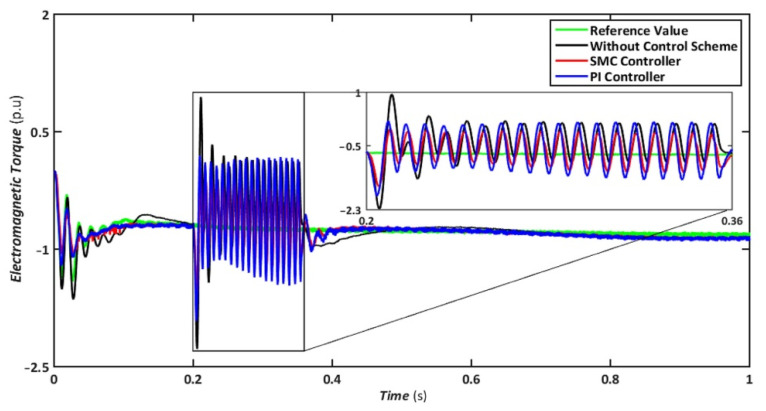
Electromagnetic torque during asymmetrical fault.

**Figure 13:**
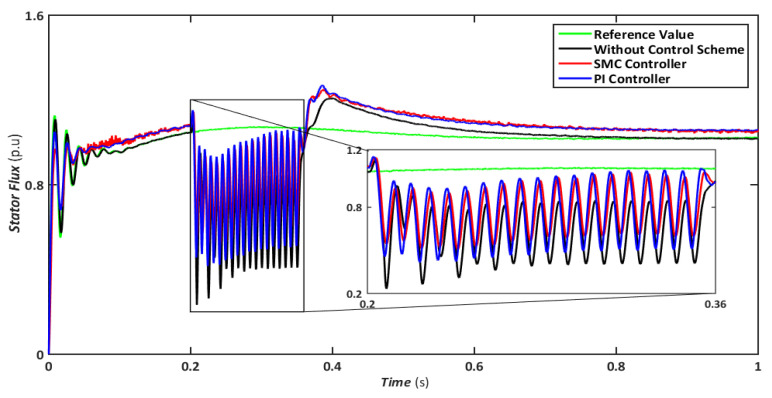
Stator flux during asymmetrical fault.

When such failures occur, which is relatively prevalent in power systems, each phase is affected negatively, resulting in several harmonic components. Consequently, the waveforms are imbalanced, resulting in the formation of large oscillations in the frequency spectrum. In this scenario, an LL-G fault is applied on a 30-km transmission line for 150 ms to evaluate the effects on various parameters. The LL-G is estimated to be responsible for 20 percent of overall power transmission failures, the second-highest cause after a line-ground fault. The effects on the DFIG essential factors and the connection between SMC and PI simulated outcomes are shown in Figure 9, Figure 10, Figure 11, Figure 12 and Figure 13. In the event of an LL-G failure, the DC-Link’s voltage approaches 1170 V for SMC and 1200 V for the PI controller, with the latter attaining slightly elevated values, as shown in Figure 9.

Nonetheless, when the system is operated without the control scheme, the voltage reaches 1390 V and fluctuates throughout the fault, as shown in Figure 10. Similarly, the voltage drops are maintained at 47, 49, and 70% for SMC, PI, and without a controller, respectively, indicating that both controllers have contributed to minimizing flux linkage disturbances. While both controllers’ rotor currents fluctuate during the LL-G fault, the SMC’s overall performance improves throughout the fault phase, as shown in Figure 11. SMC’s rotor current peaked at 0.24 pu, whereas the values for the PI controller and without a control scheme were approaching 0.29 pu and 0.39 pu, respectively. Conversely, both controllers maintained an appropriate torque amplitude, as seen in Figure 12, with SMC reaching 0.1 pu and PI attaining 0.2 pu, respectively. In contrast, the value reached positive 1.0 pu and negative 2.3 for a system without a control strategy.

Similarly, a comparison of flux amplitudes showed that the SMC and PI. Similarly, Figure 13 shows that both SMC and PI controllers experience oscillations; however, the magnitude of fluctuations is higher for PI. The proposed FRT technique can significantly control stator flux even with a negative sequence component and reduce overcurrent in the rotor circuit. Hence, the DFIG transitory response’s undamped oscillations are eliminated, and the FRT’s capabilities are enhanced.

## 5. Conclusions

This paper presents a hybrid approach combining MSFTCL with DC chopper techniques to address the problem of FRT in DFIG-based wind turbines during grid faults. The simulations are performed to assess the viability and effectiveness of the proposed system. SMC and PI controllers were used to achieve the desired performance, and the results were compared with reference values and without a control scheme. Further, two scenarios are discussed, which are (i) when the system is exposed to a balanced 3-phase symmetric fault and (ii) the most catastrophic failure on a transmission line (LL-G asymmetrical fault type). The results conclude that the proposed method supplies voltage to the reference value during symmetric and asymmetric grid failures to maintain the dynamic stability in the DFIG control system. Further, the simulation results show that the back EMF is kept to an absolute minimum, and the variations of electromagnetic torque during faults are significantly reduced. Thus, the proposed hybrid system can also regulate the reactive power efficiently while maintaining a constant DC-link voltage. Simulation results indicate that the hybrid strategy provides superior performance for all critical parameters with the advanced controller. The proposed FRT strategy is anticipated to be a major solution for improving control flexibility and operational stability in future grids with higher wind-power penetration. Moreover, the proposed strategy may also be applied to other forms of electronic power interfaced sources, such as solar energy. Although the performance of the hybrid approach has been demonstrated using the existing SMC and PI controllers, the findings may be improved further using higher-order SMC and fuzzy-PI controllers.

## Figures and Tables

**Table 1:** Parameters of DFIG.

Parameters	Values
DFIGs rated power	9 MW
Inertia constant	0.685 pu
DC-Link voltage	1150 V
Stator voltage	575 V
Mutual inductance	2.5 pu
Syn speed	2 π 60
Pitch controller gain	150
Turn ratio	1
Magnetizing resistance	0.264 Ω
Magnetizing inductance	0.0004 H
Stator-rotor leakage inductance	0.18 pu, 0.16 pu
Stator/rotor leakage resistance	0.023 pu, 0.016 pu
